# Supplementary material for: Occult Sepsis Masked by Trauma—Exploration of Cognitive Biases Through Simulation With Emergency Medicine Residents
Source: MedEdPORTAL. 2020 Nov 19;16:11023. doi: 10.15766/mep_2374-8265.11023 (PMC7678024; doi:10.15766/mep_2374-8265.11023)
Supplement: Supplementary file 1 — Case Details.docxEquipment.docxLabs and Imaging.docxDebriefing Guide.docxPostsimulation Survey.docx [file mep_2374-8265.11023-s001.zip › A. Case Details.docx]

| **Appendix A: MedEdPORTAL Simulation Case Template**  **SIMULATION CASE TITLE: Occult Sepsis Masked by Trauma**  **AUTHORS: Jonathan Weil MD, Michael Cassara DO** | |
| --- | --- |
| **PATIENT NAME: Jane Doe**  **PATIENT AGE: 68-year-old female**  **CHIEF COMPLAINT: Motor Vehicle Accident** | |
|  | |
| **Brief narrative description of case** | *Include the presenting patient chief complaint and overall learner goals for this case*  This case involves a 68-year-old female who presents after a motor vehicle collision. She arrives hypotensive and tachycardiac, with altered mental status characterized by confusion. The learners should correctly assume her abnormalities are due to hemorrhage and begin ATLS. However, the trauma evaluation fails to identify an etiology for her shock. At this point learners must reframe outside the trauma mindset, and by observing a fever and obtaining collateral information from family, recognize that the shock is due to coincident urosepsis, which caused the MVC. |
| **Primary Learning Objectives** | *What should the learners gain in terms of knowledge and skill from this case? Use action verbs and utilize Bloom’s Taxonomy as a conceptual guide*  1. Perform a primary and secondary survey as described by ATLS 10e guidelines  2. Discriminate between different types of shock  3. Identify cognitive biases and their impact on patient care |
| **Critical Actions** | *List which steps the participants should take to successfully manage the simulated patient. These should be listed as concrete actions that are distinct from the overall learning objectives of the case.*   1. Initiate fluid resuscitation immediately upon identifying hemodynamic instability 2. Obtain a finger-stick glucose 3. Complete a primary and secondary survey 4. Perform/obtain a FAST exam 5. Obtain CT imaging to rule out major sources of internal injury. 6. Obtain collateral information from the patient’s husband 7. Identify and initiate treatment for sepsis with broad-spectrum antibiotics |
| **Learner Preparation** | *What information should the learners be given prior to initiation of the case?*  *To be briefed immediately prior to entry into the room:*  “EMS has just brought in a 68-year-old female who was in a motor vehicle accident. They told us over the phone that her car struck a guard rail on the side of the road, with significant damage to the front-left side of the vehicle and airbag deployment. She was conscious but seemed confused. They started an IV and brought her straight here. No vitals reported from the field and no medications were given.”  Note: it is helpful if learners are given roughly 60 seconds after this briefing to assemble a plan of action. They can be cued that this time should be used for planning, similar to a notification prior to the arrival of a major trauma victim. |

| Initial Presentation | | | |
| --- | --- | --- | --- |
| **Initial vital signs**  **(provided only upon learner request. Displayed on wall-mounted monitor)** | T 100.6 ^o^F (38.1 ^o^C) orally P 118 BP 88/56 RR 19 Pulse ox 98% (room air) | | |
| **Overall Appearance** | *What do learners see when they first enter the room?*  An elderly female lying in a stretcher. She has a cervical collar in place. Her eyes are closed until physical stimulus is applied. She is moaning in pain. The above vital signs are already displayed on the cardiac monitor. | | |
| **Actors and roles in the room at case start** | *Who is present at the beginning and what is their role? Who may play them?*  One confederate plays the role of nurse and enters the room with the learners. The nurse draws and administers all medications and can assist with wall-mounted equipment (oxygen, suction). The nurse can also direct participants to the location of specialized equipment but does not assemble the equipment for the participants. | | |
| **HPI** | *Please specify what info here and below must be asked vs. what is volunteered by patient or other participants.*  When asked about the circumstances of the accident, the patient responds only that “I was just trying to get here…Need to be checked out…my side’s been hurting me….it’s been hurting so bad…”. Further attempts to elicit information are met by repetition of these comments. If learners persist beyond one or two attempts, the nurse confederate should suggest that “she seems very confused, I don’t think we’re going to get much more out of her.”  IF PATIENT’S HUSBAND IS CONTACTED  Husband offers that the patient was complaining of fever and right-sided back pain for the past 2 days. She was driving to her primary care doctor to get evaluated. | | |
| ***PAST MEDICAL AND SURGICAL HISTORY, MEDICATIONS, ALLERGIES, AND FAMILY HISTORY ARE PROVIDED ONLY UPON ASKING THE PATIENT’S HUSBAND*** | | |  |
| **Past Medical/Surgical History** | **Medications** | **Allergies** | **Family History** |
| Hypertension  High Cholesterol | HCTZ  Simvastatin | NKDA | None relevant |
| **Physical Examination** | | | |
| **General** | Awake, confused - oriented to person but not place or time | | |
| **HEENT** | NC/AT. Eyes open only to tactile stimuli, PERRL, EOMI. No hemotympanum. No raccoon eyes or Battle sign. No drooling, no pooling secretions. | | |
| **Neck** | Cervical collar in place. No focal tenderness. No cervical spine step-off or deformity. No bruits or expanding hematomas. | | |
| **Lungs** | CTAB | | |
| **Cardiovascular** | Tachycardiac, no m/r/g, normal S1/S2. Brisk peripheral pulses in all four extremities | | |
| **Abdomen** | Soft, non-tender, non-distended, no rebound or guarding. No blood on rectal. Positive for right CVA tenderness. | | |
| **Neurological** | Answers simple yes/no questions, otherwise does not answer appropriately, repeating the aforementioned statements. Moves all four extremities spontaneously and obeys commands. | | |
| **Skin** | Normal color for race, no wounds, no rashes | | |
| **GU** | Normal | | |

| Instructor Notes - Changes and CASE Branch Points | | |
| --- | --- | --- |
| **Intervention / Time point** | **Change in Case** | **Additional Information** |
| IV fluids or blood products ordered (timeframe: minutes 1-2) | BP improves to 100/60 after 2 minutes | If no IV fluids or blood products are ordered within two minutes the patient’s blood pressure worsens. Minimum crystalloid = 1L, Minimum blood = 2U pRBC |
| Finger stick ordered |  | Finger stick = 98 |
| Intubation | If participants elect to intubate the patient it should be done after fluid resuscitation. If the patient is intubated prior to fluids facilitators can choose whether to simply decrease the BP precipitously or to have the patient go into cardiac arrest. | Intubation is not warranted in this case, but should it be done it should be in accordance with the principles of resuscitative sequence intubation. |
| Focused Assessment with Sonography in Trauma (FAST) exam requested | RN provides images displaying negative FAST | Images available either as static frames or pre-recorded video on a tablet or monitor.  Simulated sonography may be used if available – perihepatic, perisplenic, pelvic, subxiphoid, and pulmonary views as equipment allows.  If participants request a repeat FAST several minutes after the first the results are unchanged. |
| Chest x-ray and/or pelvic xray requested | RN provides image of a normal AP chest x-ray and normal AP pelvis | Displayed via tablet or monitor |
| CT scan ordered (timeframe: by minute 10) | If sent to CT before fluid resuscitation: BP deteriorates to 60s/40s | Speed of results based on trauma activation branch point noted above.  Suggested prompt if the team fails to progress to advanced imaging: “Can we send her to the scanner to get some more data?” |
| Participants ask if any collateral sources are available (timeframe: by minute 12) | RN reports that the spouse’s phone number was found by EMS on an ICE card. RN indicates that support staff will contact the husband on behalf of the participants. | Spouse calls shortly thereafter. Information is elicited from the spouse based on what the participants ask. Spouse voice acting can be performed by an operator outside the room, calling into a telephone in the simulation bay.  Suggested prompt if team has not asked for collateral information *and* has not redirected to an alternative source of shock: “Is there anyone we can call to get some more background?” |
| Surgical service consulted | Surgical service declines intervention in the absence of an identified focal injury |  |
| BP change at minute 10 | **If the minimum fluid requirements are not given by minute 10, the BP worsens again** | Minimum volume to avoid deterioration is 2L crystalloid or 1L crystalloid + 2 units blood products or >2 units blood products |
| Antibiotics ordered | Case Ends  *If sepsis is identified early in the case, learners should still perform a comprehensive primary and secondary survey and obtain the imaging described above. Terminating the evaluation prematurely with a diagnosis of sepsis constitutes failure to achieve the critical actions* | Appropriate antibiotic coverage includes monotherapy with a broad-spectrum penicillin, cephalosporin, or carbapenem. Multidrug regimens with appropriate agents for gram-positive and gram-negative coverage would be acceptable, but are suboptimal in this patient with no allergies or risk factors for resistant pathogens. |

**Ideal Scenario Flow**

*Provide a detailed narrative description of the way this case should flow if participants perform in the ideal fashion.*

The learners enter the room to find a woman who is obviously confused with acute hemodynamic compromise after an MVC. They are unable to elicit a comprehensible history from the patient, and thus begin resuscitation on the assumption that the patient has suffered a major traumatic injury. They immediately call for either crystalloid or blood products, which produces a moderate improvement in the blood pressure. The primary survey displays the hemodynamic instability, but the patient’s mental status change is not severe enough to warrant intubation. The secondary survey is negative. The patient is deemed stable enough for CT, and a CT head/neck/chest/abdomen/pelvis is obtained given the mechanism of injury and mental status change. The CTs are negative, except for a non-specific finding of right perinephric stranding on the CT a/p. The learners contact the husband, who reports the two-day history of right sided back pain and fever. Participants then initiate further fluid resuscitation and broad-spectrum antibiotic coverage at which point the case ends.

**Anticipated Management Mistakes**

1. *Forgetting to check a point-of-care glucose:* Though not a principle objective for this module, resident teams routinely forgot to check a fingerstick glucose early in the evaluation. This critical step in the management of a confused patient became a key point in the debriefing.
2. *Delay in obtaining CT imaging:* Despite adequate fluid resuscitation and improved hemodynamics, several teams perseverated on the patient’s altered mental status as a contraindication to “transport” to radiology. The main effect was to delay their decision to obtain CT imaging. We modified the case to make a GCS > 10 more obvious based on physical exam.
3. *Anchoring on trauma:* Failure to consider how the constellation of fever, laboratory results, and CT findings was not compatible with shock related to major trauma. As expected, this served as a nidus for debriefing.
4. *Failure to obtain collateral information:* Delays in asking for collateral information left several teams uncertain about the final diagnosis and disposition. We found it helpful for the nurse confederate to suggest obtaining alternative sources of information to drive the case towards completion if a team stagnated.

**Sample Learner Assessment**

| **Critical Actions** | **Performed** | **Performed, but with partial omission or delay** | **Not Performed/Only performed with verbal cue** | **Comments** |
| --- | --- | --- | --- | --- |
| **Vitals signs** – requested within 60 seconds |  |  |  |  |
| **Fluids** - started within 2 minutes |  |  |  |  |
| **Finger stick** - obtained within 2 minutes |  |  |  |  |
| **Primary Survey** – including FAST exam |  |  |  |  |
| **Secondary Survey** |  |  |  |  |
| **CT pan-scan** -within 10 minutes |  |  |  |  |
| **Obtains Collateral Information** – within 12 minutes |  |  |  |  |
| **Identifies Septic Shock** |  |  |  |  |
| **Completes fluid resuscitation** |  |  |  |  |
| **Orders broad-spectrum antibiotics** |  |  |  |  |
