# Supplementary material for: Occult Sepsis Masked by Trauma—Exploration of Cognitive Biases Through Simulation With Emergency Medicine Residents
Source: MedEdPORTAL. 2020 Nov 19;16:11023. doi: 10.15766/mep_2374-8265.11023 (PMC7678024; doi:10.15766/mep_2374-8265.11023)
Supplement: Supplementary file 1 — Case Details.docxEquipment.docxLabs and Imaging.docxDebriefing Guide.docxPostsimulation Survey.docx [file mep_2374-8265.11023-s001.zip › B. Equipment.docx]

Appendix B: Occult Sepsis Masked by Trauma – Exploration of Cognitive Biases Through Simulation with Emergency Medicine Residents: Supply List

Personal Protective and Hygiene Equipment

- Nitrile Exam Gloves
- Face Masks/Shields (optional)
- Impervious gowns (optional)
- Alcohol-based hand soap dispenser

Medications

- Succinylcholine
- Rocuronium
- Ketamine
- Etomidate
- Epinephrine (code dose)
- Normal Saline
- Lactated Ringers
- Antibiotics (generic)

Blood Products

- Packed Red Blood Cells
- Fresh Frozen Plasma
- Prothrombin Complex Concentrate
- Platelets

Airway Equipment

- Wall-mounted oxygen flowmeter
- Wall-mounted suction canister
- Suction tubing
- Yankauer suction catheter
- Nasal cannula
- Non-rebreather
- Bag-valve mask with PEEP valve
- Laryngoscope (direct or video)
- Endotracheal tube
- Stylet
- Water-based lubricant

Point-of-Care Testing

Glucometer

Glucometer strips
