# Supplementary material for: Occult Sepsis Masked by Trauma—Exploration of Cognitive Biases Through Simulation With Emergency Medicine Residents
Source: MedEdPORTAL. 2020 Nov 19;16:11023. doi: 10.15766/mep_2374-8265.11023 (PMC7678024; doi:10.15766/mep_2374-8265.11023)
Supplement: Supplementary file 1 — Case Details.docxEquipment.docxLabs and Imaging.docxDebriefing Guide.docxPostsimulation Survey.docx [file mep_2374-8265.11023-s001.zip › C. Labs and Imaging.docx]

| Complete Blood Count (CBC) | | |
| --- | --- | --- |
| **TEST** | **VALUE** | **NORMAL RANGE** |
| WBC | 17,000 | 3,200-9,800/mm^3^ |
| HGB | 12.3 | 12-15 g/dL (female) |
|  |  | 12.6-17.2 g/dL (male) |
| HCT | 37 | 33-43% (female) |
|  |  | 39-49% (male) |
| Platelets | 270,000 | 150,000-450,000/mm^3^ |

| Basic Metabolic Panel (BMP) | | |
| --- | --- | --- |
| **TEST** | **VALUE** | **NORMAL RANGE** |
| Na | 137 | 135-145 mEq/L |
| K | 4.2 | 3.5-5 mEq/L |
| Cl | 95 | 95-105 mEq/L |
| HCO_3_ | 17 | 22-28 mEq/L |
| BUN | 20 | 8-18 mg/dL |
| Cr | 1.4 | 0.6-1.2 mg/dL |
| Glucose | 112 | 70-110 mg/dL |
| Calcium | 9.5 | 8.8-10.3 mg/dL |

| Liver Function Tests | | |
| --- | --- | --- |
| **TEST** | **VALUE** | **NORMAL RANGE** |
| AST | 32 | 0-35 Units/L |
| ALT | 16 | 0-35 Units/L |
| ALK | 98 | 30-120 Units/L |

| Amylase and Lipase | | |
| --- | --- | --- |
| **TEST** | **VALUE** | **NORMAL RANGE** |
| Lipase | 107 | 0-160 Units/L |
| Amylase | 75 | 0-130 Units/L |

| Cardiac Enzymes | | |
| --- | --- | --- |
| **TEST** | **VALUE** | **NORMAL RANGE** |
| hsT | <6 | Initial hsT <6ng/L OR Initial hsT ≥14ng/L and repeat has not changed by more than 20% |
| CPK | 115 | 20-170 Units/L |

| Venous Blood Gas | | |
| --- | --- | --- |
| **TEST** | **VALUE** | **NORMAL RANGE** |
| pH | 7.26 | 7.32-7.43 |
| PaCO_2_ | 32 | 38-55 mmHg |
| PaO_2_ | 38 | 38-42 mmHg |
| HCO_3_ | 17 | 22-26 mEq/L |
| SaO_2_ | 65 | 60-80% |
| Lactate | 5.4 | <1-2 mmol/L |

| Coagulation Studies | | |
| --- | --- | --- |
| PT | 11 | 10-13 seconds |
| INR | 1.1 | 1.0-2.0 |
| PTT | 25 | 25-35 seconds |

| Type and Screen | | |
| --- | --- | --- |
| **TEST** | **VALUE** | **NORMAL RANGE** |
| Blood Type | A | N/A |
| Rh Factor | + | N/A |

| Urinalysis (UA) | | |
| --- | --- | --- |
| **TEST** | **VALUE** | **NORMAL RANGE** |
| RBC | 0/hpf | 0-5/hpf |
| WBC | 12-25/hpf | 0-5/hpf |
| Leukocyte Esterase | Moderate | Negative |
| Specific Gravity | 1.020 | 1.010 – 1.025 |
| pH | 5.0 | 4.6 – 8 |
| Protein | Negative | Negative |
| Glucose | Negative | Negative |
| Ketones | Negative | Negative |
| Bilirubin | Negative | Negative |
| Nitrites | Negative | Negative |

**FAST**: Negative

**Chest x-ray**: Negative

**Pelvis x-ray**: Negative

**CT Head**: negative

**CT Neck**: negative

**CT Chest**: negative

**CT Abdomen/Pelvis**: non-specific perinephric stranding. Otherwise negative.
