# Supplementary material for: Occult Sepsis Masked by Trauma—Exploration of Cognitive Biases Through Simulation With Emergency Medicine Residents
Source: MedEdPORTAL. 2020 Nov 19;16:11023. doi: 10.15766/mep_2374-8265.11023 (PMC7678024; doi:10.15766/mep_2374-8265.11023)
Supplement: Supplementary file 1 — Case Details.docxEquipment.docxLabs and Imaging.docxDebriefing Guide.docxPostsimulation Survey.docx [file mep_2374-8265.11023-s001.zip › D. Debriefing Guide.docx]

Appendix D

Debriefing Guide for “Occult Sepsis Masked by Trauma – Exploration of Cognitive Biases Through Simulation with Emergency Medicine Residents”

INTRODUCTION

This guide has been prepared for faculty content experts (“debriefers”) for the simulation case scenario “Occult Sepsis Masked by Trauma.”

Emergency medicine residents spanning all levels of experience are the target audience for this simulation (“learners”). Learners have been organized into teams no greater than three members. The membership of most teams includes one senior-level resident (PGY-3 or higher) and one-or-two junior-level residents (PGY-1 and/or PGY-2). When a senior-level resident is unavailable, the membership of the team is restructured to include a combination of junior-level residents (at least one should be at the PGY-2 level). In addition to you as the content expert, you will be joined by several other faculty to assist with administration of the simulation case scenario. Additional faculty will include one simulation operations specialist (responsible for operating the technology-enhanced human mannequin simulator) and one or more simulation educators serving as “confederates” (at least one will play the role of emergency department nurse). You may be provided with a co-facilitator (simulation fellow, simulation educator, or simulation operations specialist) to assist with the debriefing.

Your role and responsibility as the session’s debriefer is to observe the learners’ performance (especially their interactions and behaviors) and assess them as the case is played. Following the conclusion of the simulation case scenario, you will lead the debrief of the learners using Advocacy-Inquiry or PEARLS (standards and methods you have learned in the Simulation Instructor Course) The tools and other information in this debriefing guide is included with the simulation case scenario as resource to help you prepare and conduct an effective debriefing.

The general flow of events for what will occur at your simulation session is diagrammed below:


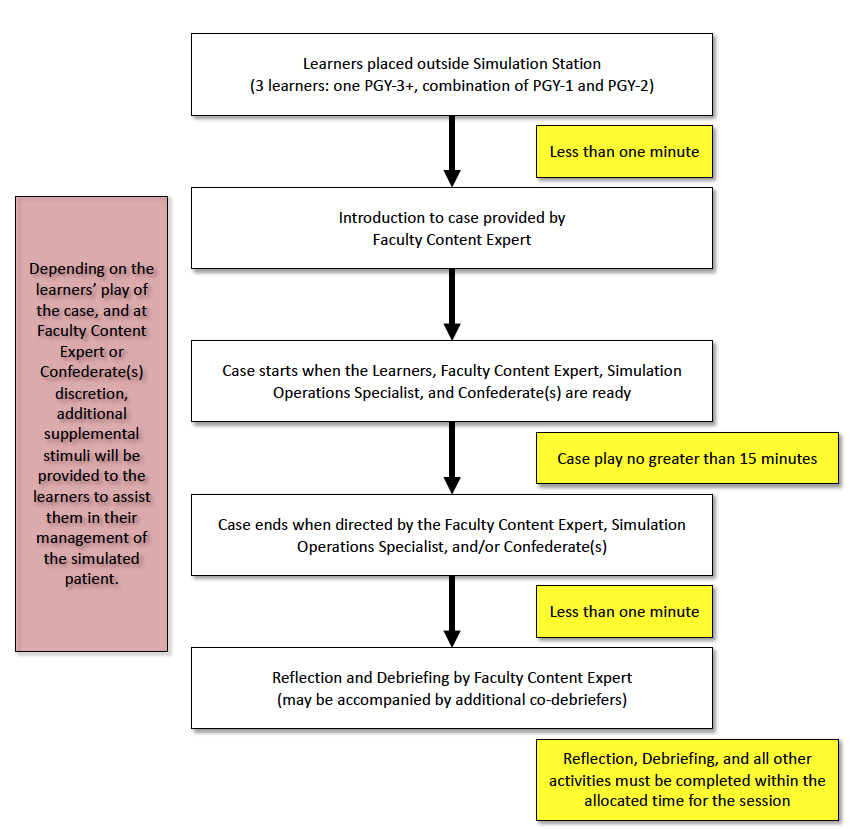


The debriefing guide has been prepared to provide you with background and reference material related to the content for the session and on the suggested debriefing method. This information has been provided to assist with the team debriefing that occurs following the play of the simulated patient encounter. Please review this content before the session. This material is not meant to replace any personal notes and material you may have, nor is it meant to be exhaustive and fully comprehensive. Please feel free to revise as necessary; additions and revisions to these notes are recommended, suggested, and encouraged. Please inform the other debriefers if you discover errors or omissions that are essential to correct, or if there is information that you believe is essential and relevant to this case that has to be included to better complete the notes within this faculty guide, so that the experience for the current learners and future participants will be enhanced.

EDUCATIONAL OBJECTIVES

1. Perform a primary and secondary survey as described by ATLS 10e guidelines
2. Discriminate between different types of shock
3. Identify cognitive biases and their impact on patient care

DEBRIEFING A SIMULATION CASE SCENARIO

This faculty guide has been created to assist debriefers and to optimize the educational experience for the learners. The debriefing is the portion of the simulation case scenario where meaningful learning is facilitated. The debriefing is classically divided into several “phases” (in the literature, there are many models; for simplicity, these have been summarized and fused into three phases: reactions, exploration, and summary):


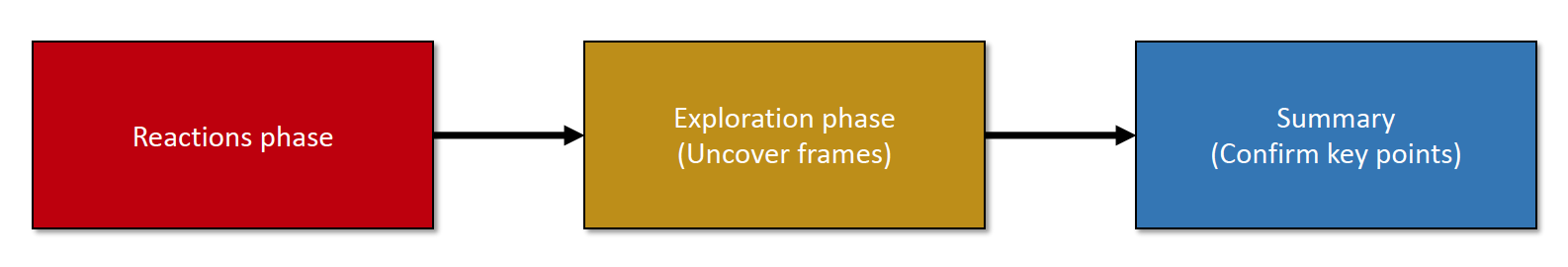


A reactions phase allows learners to acknowledge the initial lingering emotions involved with engaging in a simulation. Allowing expressions and a voice to these emotions is critical for moving into a more contemplative, objective exploration of the events which unfolded during case play. The exploration and summary phases are critical for uncovering learners’ frames and supporting or transforming them for future use. Following some of the general principles outlined below, the debriefing will be an enjoyable, engaging, interactive, and productive experience for all participants: learners, debriefers, confederates, and simulation operations specialists.

*Guiding Principles: Fundamental Belief and Psychological Safety.* Debriefers must remember to adhere to two guiding principles during the debriefing. The first guiding principle is the fundamental belief, which may be summarized in the following sentence: all learners are intelligent, caring, and capable professionals, always seeking to perform at their very highest levels, always striving to do their very best, and always embracing a growth mindset and willingness to improve.” Regardless of whether the observed performance of the learners was perfect or imperfect, debriefers must remember this guiding principle as the debriefing unfolds. Debriefers who integrate the fundamental belief establish a curious tone for the debriefing. If the observed learner performances were at optimal levels, why did that happen? If the observed learner performances deviated from the expected standard for performance, why did that happen? Debriefers embracing this guiding principle to the fullest are able to help facilitate improved future performance among learners.

To promote the honest and open exchange of ideas during the debrief, debriefers must assure learners that the learning environment is safe. The second guiding principle is psychological safety, which is defined by Lopreiato et al.^1^ as the “feeling (explicit or implicit)…that participants are comfortable participating, speaking up, sharing thoughts, and asking for help as needed without concern for retribution or embarrassment.” Debriefers who promote psychological safety encourage honest self-assessment and reflective practice among learners, through which growth and mastery are made more possible. One way to promote psychological safety, especially if paired with a co-debriefer, is to mindfully situate you and the co-debriefer among the learners within the debriefing environment to avoid establishing an implicit and unwanted dynamic, as shown in the following diagram:


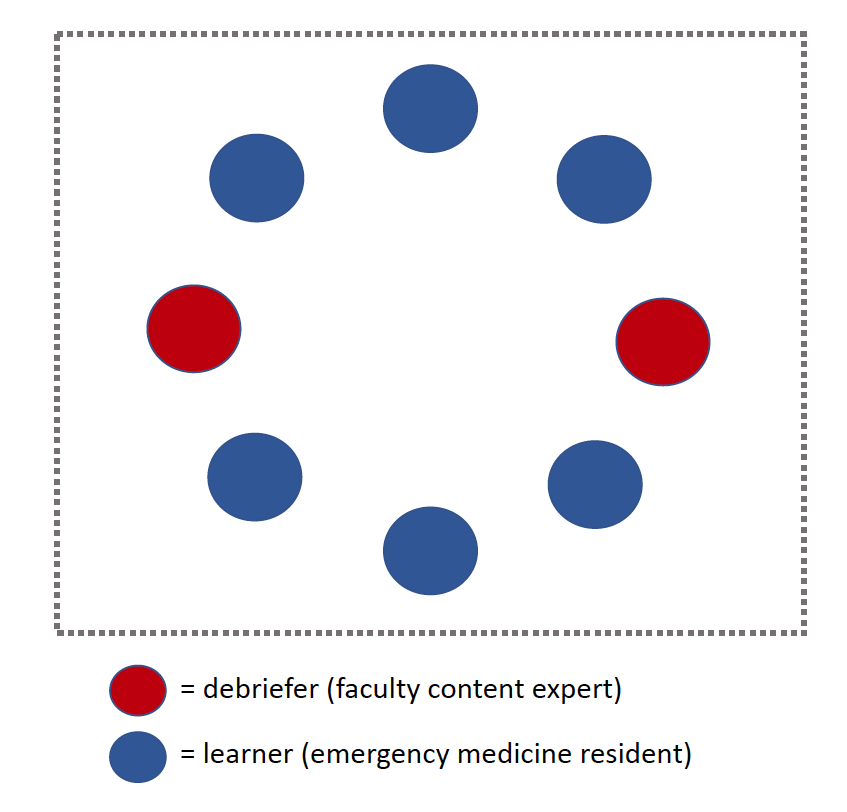


*Methods of Debriefing.* Debriefers who integrate these guiding principles into their practice are best positioned to use the debriefing models – “Debriefing with Good Judgment” ^2^ (using the process of advocacy-inquiry) or PEARLS^3^ (“promoting excellence and reflective learning in simulation”). These models align best with this simulation case scenario because of their theoretical bases in social cognitivism, social constructivism, reflective practice, experiential learning theory, meaningful learning, and principles of adult learning.

As noted earlier, both “Debriefing with Good Judgment” and PEARLS involve an initial “reactions” phase followed by a debriefer-facilitated process of open-ended questioning and exploration of the events. In “Debriefing with Good Judgment” the debriefer uses “advocacy and inquiry” as a specific questioning technique to explore the group members’ perceptions of the situation to gain a clear understanding of the groups’ (and the individual members’) “frames of mind.” As mentioned above, understanding “frames” allows debriefers to better understand why learners “did what they did” during the simulation, and provides debriefers opportunities through which they, in collaboration with the learners, can explore ways to “play it differently next time.” Examples are shown in the section below.

A feature of both of these models is that they remove the tacit, inherent expectation that faculty often feel is present when having to debrief learners: that they must be “politically correct” and that they cannot be “judgmental” or “critical” when making their assessments of the learner’s performance. Research has shown that when faculty are not clear and precise about their opinions during a debriefing that learners leave the experience confused; their behavior, consequently, remains essentially unchanged the next time they perform.

The most important aspect of debriefing with either model is the emphasis on the learner to “figure out the answers.” Faculty are coaches in these models, and are there mostly to facilitate the discussion and guide the process. If debriefing with good judgment is being performed correctly, faculty will find that they spend ≤ 25% of the debriefing time speaking, interjecting only to provide specific needed clarification and correction of misinformation when other group members do not.

References have been provided for your review and consideration. This material outlines the adult education theory and evidence-basis for using debriefing with good judgment (advocacy and inquiry) or PEARLS. Please also consider making a 30-minute investment and watch the peer-reviewed resource prepared by Hart et al.^4^, available online at <https://vimeo.com/33991081>.

CONTENT AREAS FOR DEBRIEFING, BY OBJECTIVE

1. Perform a primary and secondary survey as described by ATLS 10e guidelines

Did the team assign roles before entering a trauma resuscitation?

Optimal performance sample question phrasing: “I noticed that as a team all members decided to assign roles before entering into the case scenario, and I was curious about that, because I feel it is important to assign roles before entering situations like these. Tell me more about that.”

Suboptimal performance sample question phrasing: “I noticed that as a team all members did not assign roles before entering into the case scenario, and I was curious about that, because I feel it is important to assign roles before entering situations like these. Tell me more about that.”

Key debriefing topics:

- Discuss essential roles in a trauma resuscitation
- Describe role changes throughout a resuscitation
  - 1. How to perform a role handoff
- Consider how roles predispose to sometimes arbitrary power differentials and may impede case analysis
  - 1. Introduce concept of “authority bias”

Did the team members perform primary and secondary surveys?

Optimal performance sample question phrasing: “I noticed that the team started the case scenario by performing primary and secondary surveys, and I was curious about that, because I believe primary and secondary surveys are part of the evidence-based protocols adopted by the American College of Surgeons for trauma patient care. Tell me more about that.”

Suboptimal performance sample question phrasing “I noticed that the team did not start the case scenario by performing primary and secondary surveys, and I was curious about that, because I believe primary and secondary surveys are part of the evidence-based protocols adopted by the American College of Surgeons for trauma patient care. Tell me more about that.”

Key debriefing topics:

- Discuss standard ATLS approach of assessment: circulation, airway, breathing (CAB)
- Introduce modified approach for critical patients: C-A-C-B-C
- Abnormal vitals: awareness of tachycardia, hypotension, and fever

1. Discriminate between different types of shock

Did the team’s behavior demonstrate consideration of the causes of hypotension in a trauma patient?

Optimal performance sample question phrasing: “I noticed that all of the team members discussed the causes of hypotension in a trauma patient during the case scenario, and I was curious about that, because I believe understanding the causes of hypotension in trauma patients, and how they differ from other ED patients, is important. Tell me more about that.”

Suboptimal performance sample question phrasing: “I noticed that all of the team members did not discuss the causes of hypotension in a trauma patient during the case scenario, and I was curious about that, because I believe understanding the causes of hypotension in trauma patients, and how they differ from other ED patients, is important. Tell me more about that.”

Key debriefing topics:

- Causes of hypotension in trauma
- Principle assumption of trauma: hypotension is hemorrhage until proven otherwise
- Discuss alternative etiologies: obstructive (pneumothorax, tamponade), neurogenic
  - 1. Modalities for ruling out alternatives:
    2. Ultrasound for obstructive etiologies (E-FAST)
    3. Neuro exam – especially prior to intubation
- Essential labs: CBC, chemistry, lipase, T&S/T&C, coags
  - 1. Review disutility of H&H in the immediate post-traumatic phase
- Timing of FAST and X-rays: part of primary survey? Between primary and secondary? After secondary? Emphasize relevance to lower resources settings, e.g. fewer providers

Did the team attempt to initiate empiric treatment of the presumed most likely causes of hypotension in a trauma patient?

Optimal performance sample question phrasing: “I noticed that all of the team members started empiric treatment for hypotension in a trauma patient, and I was curious about that, because I believe starting early, empiric treatment in alignment with the evidence-based protocols adopted by the American College of Surgeons for trauma patient care, is important. Tell me more about that.”

Suboptimal performance sample question phrasing: “I noticed that all of the team members did not agree on the treatment for the patient’s hypotension, and I was curious about that, because I believe starting early, empiric treatment in alignment with the evidence-based protocols adopted by the American College of Surgeons for trauma patient care, is important. Tell me more about that.”

Key debriefing topics:

- Fluid choices in trauma
  - 1. Crystalloid vs Blood products
    2. ATLS 10e recommends no more than 1L of crystalloid
    3. Initial volume of blood products
    4. Ratios – pRBCs only vs balanced volumes in MTP (e.g. 1:1:1 pRBC:FFP:platelets)
- Venous access options for large-volume resuscitation – IO vs IV vs cordis
- Principles of sepsis resuscitation (Optional)
  - 1. Early antibiotics (< 3 hours)
    2. Volume of fluid resuscitation
       1. 3-4L before switching to vasopressors according to most trials
    3. Vasopressors of choice: vasopressin vs norepinephrine vs epinephrine

Did the team attempt to stabilize the presumed trauma patient prior to transporting the patient for advanced diagnostic imaging?

Optimal performance sample question phrasing: “I noticed that all of the team members agreed that stabilizing the patient prior to transport for advanced diagnostic imaging was a priority, and I was curious about that, because I believe early stabilization of an unstable patient prior to transport outside the ED is important and in alignment with the evidence-based protocols adopted by the American College of Surgeons for trauma patient care. Tell me more about that.”

Suboptimal performance sample question phrasing: “I noticed that the team did not (or has disagreement about) stabilizing the patient prior to transport for advanced diagnostic imaging, and I was curious about that, because I believe early stabilization of an unstable patient prior to transport outside the ED is important and in alignment with the evidence-based protocols adopted by the American College of Surgeons for trauma patient care. Tell me more about that.”

Key debriefing topics:

- Review the options for advanced diagnostic imaging when the patient is stable
  - 1. CT (with/without contrast enhancement)
- Review the options for advanced diagnostic imaging when the patient is unstable
  - 1. Point-of-care ultrasound (eFAST)
    2. Conventional XR
- Transport to radiology
  - 1. Airway security
    2. Circulatory stability
    3. Accompanying personnel in the meta-stable patient

1. Identify cognitive biases and their impact on patient care

As the case unfolded, did the team regroup and reconsider alternative hypotheses (e.g., septic shock) as results of diagnostic testing and therapeutic interventions for presumed traumatic causes of hypotension undermined and conflicted with the team’s initial assumptions?

Optimal performance sample question phrasing: “I noticed that, during the case, there was a moment when the team members stopped and reconsidered the causes for the patient’s signs and symptoms (e.g., “negative eFAST” and refractory hypotension). I am curious about that moment, because I believe it is important to continuously and objectively reevaluate our assumptions as we obtain results to our diagnostic tests and therapeutic interventions that are contrary to what we would expect. Tell me more about that.”

Suboptimal performance sample question phrasing: “I noticed that, during the case, there was a moment when the team members seemed confused about the causes for the patient’s signs and symptoms but kept following the original “gameplan.” I am curious about that moment, because I believe it is important to continuously and objectively reevaluate our assumptions as we obtain results to our diagnostic tests and therapeutic interventions that are contrary to what we would expect. Tell me more about that.”

Key debriefing topics:

- Cognitive Biases
- Heuristics as the starting point for trauma resuscitation
  - 1. Introduce the definition of a heuristic
    2. Identify heuristics in trauma – “hypotension is bleeding until proven otherwise,” “ABCs/CABs”
    3. Discuss the value of heuristics – they tend to rapidly focus our attention on the most common or most serious pathologies
    4. When do heuristics mislead us?
       1. Uncommon presentations of common conditions
       2. Rare conditions we haven’t seen before
       3. “Great imitator” pathologies – e.g. classically syphilis, but pulmonary embolism might be a better modern example
- Major biases to consider in a trauma resuscitation
  - 1. Authority Bias
       1. Tendency to trust an assessment or recommendation because it comes from a position of power
       2. Risk inherent in any hierarchical situation – e.g. trauma team leader assumes responsibility; a resident reports to an attending; a medical student reports to a resident
    2. Anchoring Bias and Diagnostic Momentum
       1. Tendency to continue down an analytical or treatment pathway based on a few features of the case, but failing to consider alternatives when appropriate
       2. Labels as a driving force
          1. Triage labels
          2. Prior diagnostic labels
       3. Search Satisfying
          1. Failure to evaluate for other pathologies when the first plausible explanation is found
          2. “The most commonly missed fracture is the second fracture”
- How do we overcome these biases?
  - 1. Cognitive forcing strategies
       1. A strategy designed to reframe or challenge a thought process or conclusion
       2. Common example in EM: “worst first” consideration before settling on a benign diagnosis
- Checklists
  - 1. Trauma evaluation checklist
    2. Procedural checklist
    3. Sedation/Airway checklist
- Devil’s Advocate/Red team
  - 1. An external team or individual assigned to find fault or force consideration of alternative strategies
    2. Can challenge on an individual, departmental, or systems level
    3. An individual can replicate this by operating on the assumption that their first conclusion is wrong and forcing themselves to re-evaluate.

REFERENCES

1. Lopreiato JO. *Healthcare simulation dictionary.* Agency for Healthcare Research and Quality; 2016.
2. Rudolph J, Simon R, Dufresne R, Reamer D. There is no such thing as non-judgemental debriefing: a theory and method for debriefing with good judgement. Simul Healthc. 2006; 1: 49–55.
3. Eppich W, Cheng A. Promoting Excellence and Reflective Learning in Simulation (PEARLS): development and rationale for a blended approach to health care simulation debriefing. *Simulation in Healthcare.*2015; 10(2):106-115.
4. Hart D, McNeil MA, Griswold-Theodorson S, Bhatia K, Joing S. High fidelity case-based simulation debriefing: everything you need to know. *Acad Emerg Med.*2012; 19(9):E1084.

A Primer on Heuristics and Cognitive Bias

*The following is meant to serve as a brief introduction to heuristics and their potential shortcomings. It can be used as pre- or post-reading by learners, or as the basis for a didactic session. The language is intentionally simplified to remain accessible to the learner. It is adapted from the following references noted in the ESR - Kahneman (2011), Saposnik (2016), Graber (2012), and Sherbino (2014).*

*What is a Heuristic?*

When approaching a clinical problem, experienced clinicians rarely follow a discrete analytic process that connects all their observations in order to determine a diagnosis or treatment. Nonetheless, they come to the correct answer the vast majority of the time. The classic example of this is the wizened doctor who walks into a room and makes the diagnosis from the foot of the bed almost instantaneously. We accomplish this through heuristics – “mental shortcuts,” so to speak, that allow us to make rapid decisions by relating the current situation to mental models developed through prior experiences. We quickly categorize the current clinical encounter not by conscientiously taking stock of every piece of data, but instead by subconsciously drawing connections between what we see now and something we have seen before.

*Heuristics in Everyday Life*

We succeed, to varying degrees, in our daily activities as a function of the heuristics we have developed for nearly every activity in our daily lives. They provide fundamental and rapidly accessible assumptions about what we can expect in a situation and how we should conduct ourselves or react. Researchers like to draw our attention to how useful heuristics have been throughout human history. A prototypical example is how we would react to hearing a growling sound come out of a dark cave, or any other situation that would elicit fear. A person would not need to analyze the nature of the growling sound, its precise intonation, volume, and duration, followed by a careful examination of the dark cave, before concluding that they should leave the area. Such a fundamental survival heuristic allows us to make the split-second decision by comparing the current situation to previous experiences, and concluding that, in the past, nothing good has come from venturing into caves with loud growling noises, and therefore we should not do so here. Conveniently, however, this gets at another key feature of heuristics, namely that they can be wrong. The growling sound could be from a creature that is small compared to even a petite person, its vocalizations incidentally amplified by the acoustics of the cave. We might be in no danger whatsoever. But for most people, our mental models do not make these kinds of nuanced discriminations. We categorize and thereby respond to a situation instantaneously, without any conscious thought. That is not to say that we are slaves to them, but we are all *susceptible* not them. Ultimately, it is for a good reason in many cases. The vast majority of the time, it *is* ill-advised to walk into that cave.

*What do we mean by “previous experiences”?*

Heuristics develop from our assessments, conscious or otherwise, of prior experiences. By necessity, we can only deploy a heuristic if the situation we face bears some relationship to a scheme we have developed in our minds. However, “experience” does not necessarily depend on having physically faced an identical situation, or even one that bears a resemblance. For instance, stereotypes are a heuristic, but they can arise without ever having interacted with the stereotyped group. Hearing how friends, family, coworkers, or the media we consume address a particular ethnic group will lead us to develop schemas for how to act when we encounter them. However, this is arguably the most prominent, modern day example of how heuristics lead us astray. Stereotypes induce snap categorizations about a person based on a religious or ethnic affiliation (to name but a few), but they may not apply to the individual or they may be incorrect altogether. Thus, heuristics arise from experience, but are only valuable to us if the experience is representative of the reality we face.

*Examples of Heuristics in Medicine*

Instructors probably taught you heuristics explicitly in the past, but they were more likely referred to as “rules of thumb.” Consider the sentiment that hypotension in trauma is bleeding until proven otherwise. This is a heuristic. It is a model for how to approach a clinical situation. It is useful for focusing our evaluation to identify the most commonly lethal pathology in traumatic injuries. It may also be wrong. Some patients will have hypotension from an alternative etiology, such as a pneumothorax or cardiac tamponade, and blind adherence to this rule of thumb would have grave consequences. Thus, our ability to use it effectively hinges on awareness of its limitations at the same time that we use it to make rapid decisions about the direction of our care.

*Cognitive Biases*

Judgement errors arising from heuristics – such as stereotyping – are broadly categorized as “cognitive biases.” The bias in question is any factor that leads us to prematurely conclude our investigations. In this sense of the word, “bias” refers to our existing heuristics. However, as mentioned previously, heuristics are largely born from some form of experience. As a result, what we label as bias may actually represent a shortage of data to fully inform our schema, i.e. a shortage of experience. For instance, acute cholecystitis can present primarily as flank pain, easily confused for a pattern more reminiscent of nephrolithiasis. The clinician who has seen more cases of flank pain compared to a junior colleague will have a better developed heuristic and might make the diagnosis faster. However, no heuristic can guarantee perfection, and there will always be limits to how much time we have available to study or practice. Thus, we define the following cognitive biases with the aim of then considering the tools at our disposal to avoid them.

*A Few Types of Cognitive Bias*

The following represent a few examples of cognitive bias, not a comprehensive list.

Anchoring Bias - The tendency to adhere to a conclusion based on a few salient facts, without considering how other information, available concurrently or later in the case, changes the clinical picture. A common example of anchoring arises from triage notes swaying a clinician’s thought process, especially when signaling features that draw on social stereotypes such as a history of mental illness or drug use.

Authority Bias - Agreement with a decision principally because it was suggested by someone we view as a superior. It can arise because we inherently trust the person or because we are afraid to contradict an apparent authority and risk private or public humiliation.

Search Satisfying – Prematurely terminating our evaluation because we have found an explanation for the symptoms, without considering that there may be more than one underlying pathology

The exact label given to a heuristic failure is less important than recognizing that we are universally susceptible to them.

*Potential Solutions*

Any tool that forces us to slow down and contemplate how we arrived at a decision may avoid a cognitive error. Perhaps without knowing it, the medical profession has made some of the strategies for counteracting cognitive bias a part of its DNA. For instance, the differential diagnosis is a scheme for broadening our thought process when applied to cases where the diagnosis seems obvious. Some clinicians choose not to look at the triage note before evaluating a patient. A team leader might ask for input from the team members before giving her own opinion. Broadly speaking, we can introduce checks into our thought processes or change the way we interact with the environment. The former depends on the motivation or expertise of the individual, while the latter may impact clinicians with no knowledge of heuristic failures.
