# Supplementary material for: Occult Sepsis Masked by Trauma—Exploration of Cognitive Biases Through Simulation With Emergency Medicine Residents
Source: MedEdPORTAL. 2020 Nov 19;16:11023. doi: 10.15766/mep_2374-8265.11023 (PMC7678024; doi:10.15766/mep_2374-8265.11023)
Supplement: Supplementary file 1 — Case Details.docxEquipment.docxLabs and Imaging.docxDebriefing Guide.docxPostsimulation Survey.docx [file mep_2374-8265.11023-s001.zip › E. Postsimulation Survey.docx]

**Participant Impression Survey**

Overall, how satisfied or dissatisfied were you with this case?

| Very dissatisfied | Somewhat dissatisfied | Neutral | Somewhat satisfied | Very satisfied |
| --- | --- | --- | --- | --- |

How much did this case contribute to your understanding of advanced trauma life support (ATLS)?

| Not at all | A little | A moderate amount | A lot | A great deal |
| --- | --- | --- | --- | --- |

How much did this case contribute to your understanding of sepsis management?

| Not at all | A little | A moderate amount | A lot | A great deal |
| --- | --- | --- | --- | --- |

How much did this case contribute to your understanding of cognitive biases, such as anchoring bias?

| Not at all | A little | A moderate amount | A lot | A great deal |
| --- | --- | --- | --- | --- |

What is your current year of training?

| PGY-1 | PGY-2 | PGY-3 | PGY-4 or Above |
| --- | --- | --- | --- |
