# Supplementary material for: Occult Sepsis Masked by Trauma—Exploration of Cognitive Biases Through Simulation With Emergency Medicine Residents
Source: MedEdPORTAL. 2020 Nov 19;16:11023. doi: 10.15766/mep_2374-8265.11023 (PMC7678024; doi:10.15766/mep_2374-8265.11023)
Supplement: Supplementary file 1 — Case Details.docxEquipment.docxLabs and Imaging.docxDebriefing Guide.docxPostsimulation Survey.docx [file mep_2374-8265.11023-s001.zip › _Educational_Summary_Report_11023.pdf]

MedEdPORTAL® The AAMC Journal of Teaching and Learning Resources

Original Publication Open Access

Occult Sepsis Masked by Trauma—Exploration of Cognitive Biases Through Simulation With Emergency Medicine Residents

Jonathan Weil, MD*, Michael Cassara, DO, MSEd

*Corresponding author: weil.jon@gmail.com

Abstract

Introduction: There is a paucity of simulation literature and curricula addressing cognitive bias and the skills necessary to overcome this common source of clinical error. We designed a scenario for emergency medicine (EM) residents with the intent to trigger an anchoring bias as a nidus for conversation about metacognition. **Methods:** We implemented this case for teams of two to three PGYs 1-5, including both EM and EM/internal medicine residents within a longitudinal simulation curriculum. The case was designed to simulate a major trauma wherein evaluation according to standard advanced trauma life support principles failed to identify a traumatic injury to explain the patient's hemodynamic instability. Residents had to reorient their thought process towards other etiologies of shock, ultimately identifying sepsis as the driving force behind the trauma. The scenario ran over 10-15 minutes followed by a 30-minute debrief. Case satisfaction and the success of various learning objectives were assessed via a postsimulation survey. **Results:** Forty-four EM and combined EM/IM residents ranging from PGY 1-5 participated in the simulation over a 5-week period. Nearly 82% of respondents expressed an overall satisfaction with the case. About 76% felt the case succeeded in contributing to their understanding of cognitive biases. **Discussion:** The implementation of misdirection in this simulation was an effective means by which to engage learners in education about cognitive biases.

Keywords
Medical Simulation, Cognitive Bias, Sepsis, Trauma, Physician, Clinical Reasoning/Diagnostic Reasoning, Emergency Medicine, Simulation

Educational Objectives

By the end of this activity, learners will be able to:

1. Perform a primary and secondary survey as described by standard advanced trauma life support guidelines.
2. Discriminate between different types of shock.
3. Identify cognitive biases and their impact on patient care.

Introduction

Though the importance of medical error in patient morbidity and mortality has been established for decades, the relative contribution of various errors and the means to mitigate their impact remains a topic of substantial debate. Cognitive error is one such subdomain, with a robust body of literature describing its omnipresence in general and in clinical decision-making, yet there remains a dearth of strategies for effectively addressing it.[1-6] In fact, methods for mitigating bias (i.e., debiasing) and thereby yielding a meaningful change in clinical behavior are extremely sparse.[7] Medical educators should, at a minimum, seek to educate learners about the field of metacognition, yet even here many curricula fall short.[8] The net effect, then, is that even if a curriculum is suitably inclusive of the cognitive bias concept, traditional teaching methods have little to offer in terms of translatable clinical outcomes. However, it remains to be seen if simulation is a viable methodology in this realm. There are a limited number of studies assessing its viability.[9] Prior works demonstrated the value of both low- and high-fidelity exercises as mediums for exploring and reproducing cognitive biases.[10,11]

Citation:
Weil J, Cassara M. Occult sepsis masked by trauma—exploration of cognitive biases through simulation with emergency medicine residents. *MedEdPORTAL*. 2020;16:11023.
https://doi.org/10.15766/mep_2374-8265.11023

Within *MedEdPORTAL* there was a paucity of simulation experiences that dealt directly with cognitive biases, with a small number oriented specifically towards emergency medicine (EM) trainees.[12-16] This represented a clear deficit in educational resources given the aforementioned interest in metacognitive skills in recent decades. Furthermore, the uniquely demanding clinical environment of EM, with its combination of high throughput caseload, time sensitivity, and high acuity, likely places practitioners at risk for heuristic-based errors in clinical judgement. We therefore designed a case for EM residents with

Copyright © 2020 Weil and Cassara. This is an open-access publication distributed under the terms of the Creative Commons Attribution license.

the goal of inducing an anchoring bias in the setting of major trauma to act as a frame for discussion about cognitive biases. To our knowledge, there is no other published case that explores this domain in the setting of trauma. Since the standard of care in trauma management is among the most algorithmic schemas in EM it potentially predisposes to such cognitive biases.

Methods

We developed this case as part of the longitudinal simulation curriculum for EM residents at the joint North Shore University and Long Island Jewish Hospitals program. PGYs 1-5, including both EM and EM/internal medicine (IM) residents, participated in weekly simulation scenarios. The typical structure was 10-12 residents broken into teams of two to three, consisting of a senior resident (PGY 3 or above) and one to two junior residents. Each team participated in a simulation session lasting up to 15 minutes followed by a 25-35 minute debrief (Appendix A). With regards to this case, all learners were certified in advanced trauma life support (ATLS). However, they were presumed to have drastically different real-world experience with trauma based on their year of training, as exposure to such cases at our institutions increases exponentially in PGY 2 and above. All residents had a fundamental knowledge of sepsis management.

Equipment/Environment

We ran this case in the Northwell Patient Safety Institute, which is the dedicated simulation facility for the residency program. We used the SimMan 3G (Laerdal Medical Corporation) prepped with a single large-bore IV and wig. No additional moulage was used. Continuous cardiac telemetry, pulse oximetry, and blood pressure cuff were available, with data displayed on a single monitor upon request. The room also contained standard wall-mounted resources such as oxygen, air, and suction access points. A mock code cart was stocked with resuscitative equipment—supplemental oxygen devices, advanced airway tools, and defibrillator—and medications (Appendix B). All labs and imaging were presented on a tablet (Appendix C).

Personnel

Teams were structured as described above. All residents entered the room simultaneously upon case initiation. A nurse confederate delivered the prebrief, was present to administer all medications, and provided predefined cues (Appendix A). SimMan 3G was operated by dedicated simulation lab personnel in conjunction with an attending physician, though this could be performed by anyone with basic operating knowledge of the mannequin. Changes in vital signs or mannequin physical exam features were at the discretion of the attending physician, guided by the scenario script.

Implementation

Before entering the room, we provided each team a report from emergency medical services regarding the circumstances of the trauma victim, described as an elderly female in a single-car collision. They were informed the patient now had IV access but received no interventions. They entered the room to find an obviously confused woman with acute hemodynamic compromise after a motor vehicle collision. Due to the patient's confusion, participants were unable to elicit any additional history from the patient. Optimal management was resuscitation on the assumption that the patient suffered a major traumatic injury, starting with provision of either crystalloid or blood products, which produced a moderate improvement in the hemodynamics. Failure to provide fluids or blood early enough resulted in further deterioration. The primary survey displayed the hemodynamic instability, but the patient's mental status change was not severe enough to warrant intubation. The secondary survey was negative. At this point, residents had to determine if the patient was stable enough for CT. Further deterioration ensued if the patient was sent to radiology prior to receiving an adequate volume of fluid. The case was fast-forwarded after the patient was sent to CT, with imaging results provided immediately. The CTs were negative except for a nonspecific finding of right perinephric stranding on the CT abdomen/pelvis. Labs were significant mainly for a leukocytosis and lactic acidosis, but not inherently diagnostic (Appendix C). Upon request for collateral, the learners were provided contact information for the patient's husband, who reported a 2-day history of right-sided back pain and fever. Participants then initiated further fluid resuscitation and broad-spectrum antibiotic coverage, which ended the case. If learners were unable to reach this decision by minute 15 the confederate or attending physician asked for a brief case summary and disposition decision to terminate the encounter.

Debriefing

Participants debriefed for 25-35 minutes immediately following the simulation. The primary facilitator was an attending physician who was fellowship-trained in simulation, or one who had completed the Northwell Health Patient Safety Institute simulation leadership course. They were occasionally assisted by a medical simulation fellow or senior resident (PGY 3 or above). The debriefing format primarily followed either the advocacy-inquiry or promoting excellence and reflective learning in simulation (PEARLS) modalities.[17,18] A sample format was provided in Appendix D.

Assessment

Teams were assessed formatively based on a critical action checklist (Appendix A). We designed this checklist to encapsulate

Copyright © 2020 Weil and Cassara. This is an open-access publication distributed under the terms of the Creative Commons Attribution license.

key elements of ATLS management, (primary and secondary survey, bedside imaging, helical imaging, and laboratory studies), sepsis management (volume resuscitation, empiric antibiotics), and general evaluation of the altered patient (blood glucose level, obtaining collateral information). Residents evaluated the effectiveness of the simulation in achieving its learning objectives via an online survey (SVMK Inc; Appendix E).

Results

Forty-four EM and combined EM/IM residents ranging from PGY 1-5 participated in the simulation over a 5-week period.

Of residents, 21 out of 44 (47%) responded to the postsimulation survey. The survey assessed attitudes towards the simulation experience on a series of 5-point Likert scales (1 = *very dissatisfied*, 5 = *very satisfied*; 1 = *not at all*, 5 = *a great deal*). Of those, 17 (81%) described their experience with the simulation as either somewhat (4) or very satisfying (5). Most respondents thought the case added modestly to their knowledge of sepsis and trauma management. Specifically, 17 (81%) thought it added *a moderate amount* (3) or *a lot* (4) to their understanding of ATLS, while 13 (62%) said the same about sepsis. With regards to the primary learning objective, most respondents reported that the case added to their understanding of cognitive bias, with 16 (76%) stating they learned *a lot* (4) or *a great deal* (5) about this topic.

Discussion

To address a gap in simulation-related learning resources related to cognitive bias in high-acuity settings and a general paucity of focus on this topic in resident education, we developed a simulated resuscitation case designed to induce anchoring tendencies.

This case proved to be an effective stimulus for discussing cognitive bias, with a large majority of residents reporting high satisfaction. As expected, the value of the trauma and sepsis education derived from this case was modest, as the ATLS evaluation was standard by design and the required sepsis treatment uncomplicated. As we first deployed this late in the academic year even learners at an early stage in training had a basic knowledge of both. The perceived value derived from the emphasis on metacognition outstripped that from either trauma or sepsis, with responses skewing more towards learning *a lot* or *a great deal*. This case could be a useful means of teaching basic trauma resuscitation and sepsis management, but we expect that, especially for senior residents, it was most valuable for discussions beyond shock.

One challenge encountered early in development was maintaining learner buy-in to the scenario. Suspension of disbelief is a prerequisite for any simulation, but during the debriefing we encountered more resistance than is typical at our institution. Subjectively, this was more pronounced with an earlier draft of the case when the patient was younger and the fever less evident. Revising the patient age upwards and increasing the core temperature helped resolve some concerns that the scenario was not realistic, which proved to be a reasonable compromise for maintaining engagement in the debriefing. Resistance still remained a theme among some groups, particularly if they had difficulty identifying the underlying sepsis; however, it also provided an excellent opportunity to quickly make cognitive bias the centerpiece of the discussion. Overall from our observations most participants, especially relatively senior residents, appreciated the diagnosis twist, as it was sometimes referred to.

The major limitation to the collected data was the small sample size and low response rate, the latter likely arising from bulk distribution of the survey after all residents in the cohort had experienced the simulation. This raised the prospect of biased results in the descriptive data. For instance, one can imagine that participants who were less pleased with their experience might have a lower response rate. The data could also be improved with presimulation surveys on awareness of cognitive bias; however, we excluded this out of concern that it would change how residents approached the case. This might be avoided by either doing a remote pretest weeks or months before, clustering questions with other pretest assessments, or using nonparticipant residents as a control group to assess knowledge of cognitive bias. Future deployments of this simulation using these techniques will provide a better understanding of how it changes resident awareness of metacognitive skills. It should also be noted that this case did not assess if simulation resulted in a clinically relevant change in this skill set. Results were isolated to Kirkpatrick level 1 evaluation.

In summary, this case was the first in the *MedEdPORTAL* database to combine an occult etiology of shock, a high-risk and time-sensitive pathology, with an exploration of cognitive biases. It provided a useful framework for discussing various cognitive biases, as well as discussing the fundamentals of trauma and sepsis management.

Appendices

A. Case Details.docx

B. Equipment.docx

Copyright © 2020 Weil and Cassara. This is an open-access publication distributed under the terms of the Creative Commons Attribution license.

C. Labs and Imaging.docx

D. Debriefing Guide.docx

E. Postsimulation Survey.docx

All appendices are peer reviewed as integral parts of the Original Publication.

Jonathan Weil, MD: Resident, Department of Emergency Medicine, North Shore University/Long Island Jewish Hospitals; ORCID: https://orcid.org/0000-0001-6511-0444

Michael Cassara, DO, MSEd: Associate Professor, Department of Emergency Medicine, Zucker School of Medicine; Medical Director, Patient Safety Institute and Emergency Medical Institute, Northwell Health

---

Disclosures
None to report.

Funding/Support
None to report.

Ethical Approval
Reported as not applicable.

References

1. Kohn KT, Corrigan JM, Donaldson MS. *To Err is Human: Building a Safer Health System*. National Academy Press; 1999.
2. Kahneman D. *Thinking, Fast and Slow*. Farrar, Straus and Giroux; 2011.
3. Sherbino J, Norman GR. Reframing diagnostic error: maybe it's content, and not process, that leads to error. *Acad Emerg Med*. 2014;21(8):931-933. https://doi.org/10.1111/acem.12440
4. Graber ML, Franklin N, Gordon R. Diagnostic error in internal medicine. *Arch Intern Med*. 2005;165(13):1493-1499. https://doi.org/10.1001/archinte.165.13.1493
5. Saposnik G, Redelmeier D, Ruff CC, Tobler PN. Cognitive bias associated with medical decisions: a systematic review. *BMC Med Inform Decis Mak*. 2016;16:138. https://doi.org/10.1186/s12911-016-0377-1
6. O'Sullivan ED, Schofield SJ. Cognitive bias in clinical medicine. *J R Coll Physicians Edinb*. 2018;48(3):225-232. https://doi.org/10.4997/jrcpe.2018.306
7. Smith BW, Slack MB. The effect of cognitive debiasing training among family medicine residents. *Diagnosis*. 2015;2(2):117-121. https://doi.org/10.1515/dx-2015-0007
8. Rencic J, Trowbridge RL Jr, Fagan M, Szauter K, Durning S. Clinical reasoning education at US medical schools: results from a national survey of internal medicine clerkship directors. *J Gen Intern Med*. 2017;32(11):1242-1246. https://doi.org/10.1007/s11606-017-4159-y
9. Graber ML, Kissam S, Payne VL, et al. Cognitive interventions to reduce diagnostic error: a narrative review. *BMJ Qual Saf*. 2012;21(7):535-557. https://doi.org/10.1136/bmjqs-2011-000149
10. Prakash S, Bihari S, Need P, SPric C, Schuwirth L. Immersive high-fidelity simulation of critically ill patients to study cognitive errors: a pilot study. *BMC Med Educ*. 2017;17(1):36. https://doi.org/10.1186/s12909-017-0871-x
11. Park CS, Stojiljkovic L, Milicic B, Lin BF, Dror IE. Training induces cognitive bias: the case of a simulation-based emergency airway curriculum. *Simul Healthc*. 2014;9(2):85-93. https://doi.org/10.1097/sih.0b013e3182a90304
12. Denson K, Manzi G, Rehm J, Malmsten C. Geriatric cardiology OSCE: the hidden curriculum, identifying hypoactive delirium when obtaining procedural consent. *MedEdPORTAL*. 2013;9:9601. https://doi.org/10.15766/mep_2374-8265.9601
13. Hunzeker A, Amin R. Teaching cognitive bias in a hurry: single-session workshop approach for psychiatry residents and students. *MedEdPORTAL*. 2016;12:10451. https://doi.org/10.15766/mep_2374-8265.10451
14. Paolo W. Medical heuristics and cognitive decision errors: modeling thought processes through an integrative curriculum. *MedEdPORTAL*. 2013;9:9375. https://doi.org/10.15766/mep_2374-8265.9375
15. Chew K, van Merrienboer J, Durning S. Teaching cognitive biases in clinical decision-making: a case-based discussion. *MedEdPORTAL*. 2015;11:10138. https://doi.org/10.15766/mep_2374-8265.10138
16. Shapiro M, Kobayashi L, Overly F, et al. CMS transportable simulation-based training curriculum in patient safety. *MedEdPORTAL*. 2009;5:592. https://doi.org/10.15766/mep_2374-8265.592
17. Rudolph JW, Simon R, Raemer DB, Eppich WJ. Debriefing as formative assessment: closing performance gaps in medical education. *Acad Emerg Med*. 2008;15:1010-1016. https://doi.org/10.1111/j.1553-2712.2008.00248.x
18. Eppich W, Cheng A. Promoting excellence and reflective learning in simulation (PEARLS): development and rationale for a blended approach to health care simulation debriefing. *Simul Healthc*. 2015;10(2):106-115. https://doi.org/10.1097/sih.0000000000000072

Received: April 3, 2020
Accepted: July 8, 2020
Published: November 19, 2020

Copyright © 2020 Weil and Cassara. This is an open-access publication distributed under the terms of the Creative Commons Attribution license.
